# Supplementary material for: Selective posttranslational inhibition of CaVβ1-associated voltage-dependent calcium channels with a functionalized nanobody
Source: Nat Commun. 2022 Dec 9;13:7556. doi: 10.1038/s41467-022-35025-7 (PMC9734117; doi:10.1038/s41467-022-35025-7)
Supplement: Supplementary file 1 — Supplementary Information [file 41467_2022_35025_MOESM1_ESM.pdf]

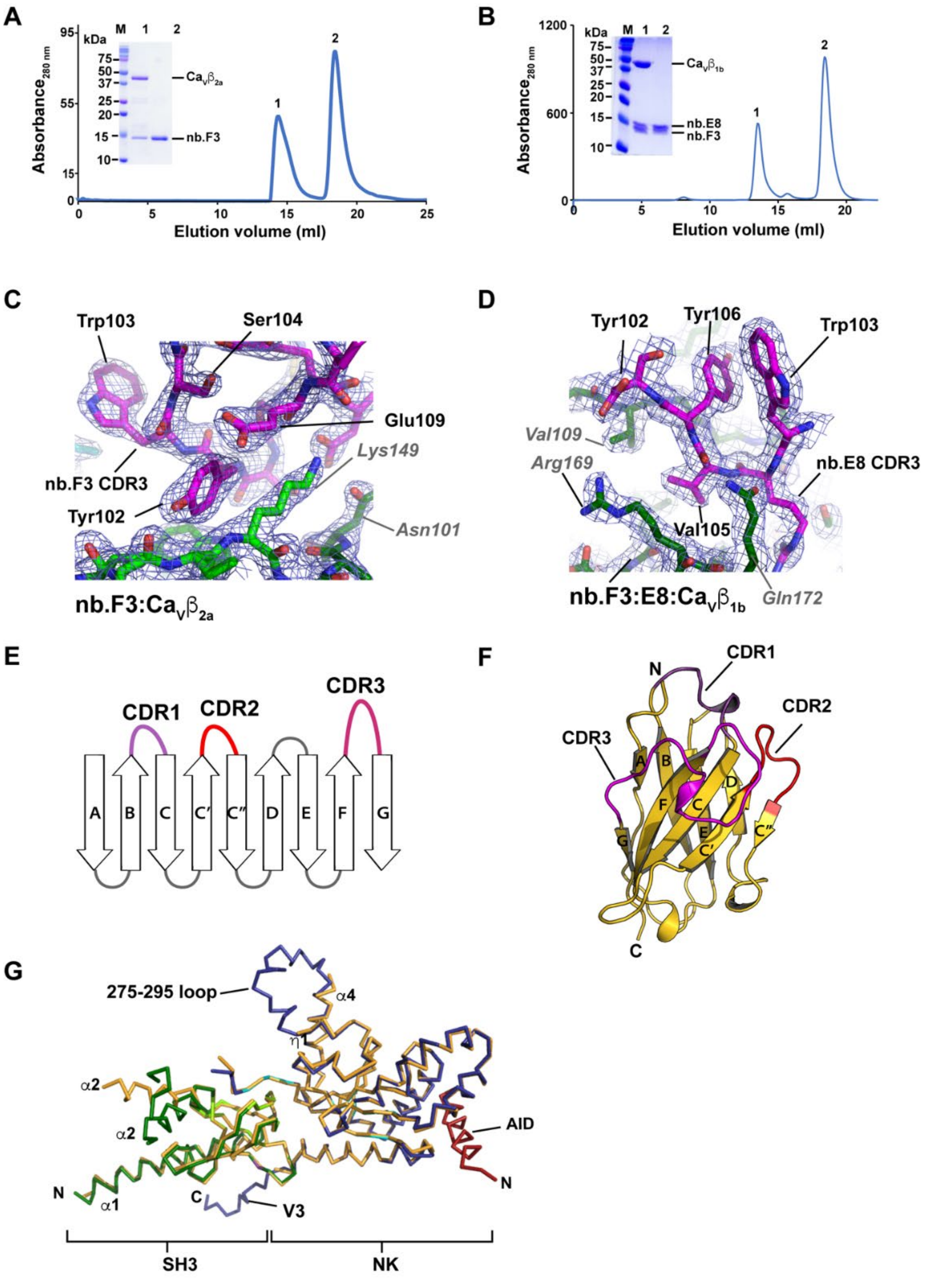

Supplementary Figure 1: nanobody:Ca$_V$β complex biochemical and structural characterization. A and **B,** Size exclusion chromatography (Superdex 200) for **A,** nb.F3:Ca$_V$β$_{2a}$ and **B,** nb.F3:nb.E8:Ca$_V$β$_{1b}$. Exemplar electron density of residues at interface between **C,** nb.F3 (magenta) and Ca$_V$β$_{2a}$ (green) **D,** nb.E8 (magenta) and Ca$_V$β$_{1b}$ (green). **E,** Nanobody schematic. CDR1 (purple), CDR2 (red), CDR3 (magenta), and β-strands are labeled. **F,** Cartoon diagram of nb.F3 from the nb.F3:Ca$_V$β$_{2a}$ complex. CDRs are colored as in 'E'. **G,** Superposition of Ca$_V$β$_{2a}$ (blue and green) from the nb.F3:Ca$_V$β$_{2a}$ and Ca$_V$β$_{2a}$ (yellow orange) from the Ca$_V$β$_{2a}$:AID (PDB:5V2P)[1] complex. AID is firebrick. Select Ca$_V$β$_{2a}$ elements are labeled.

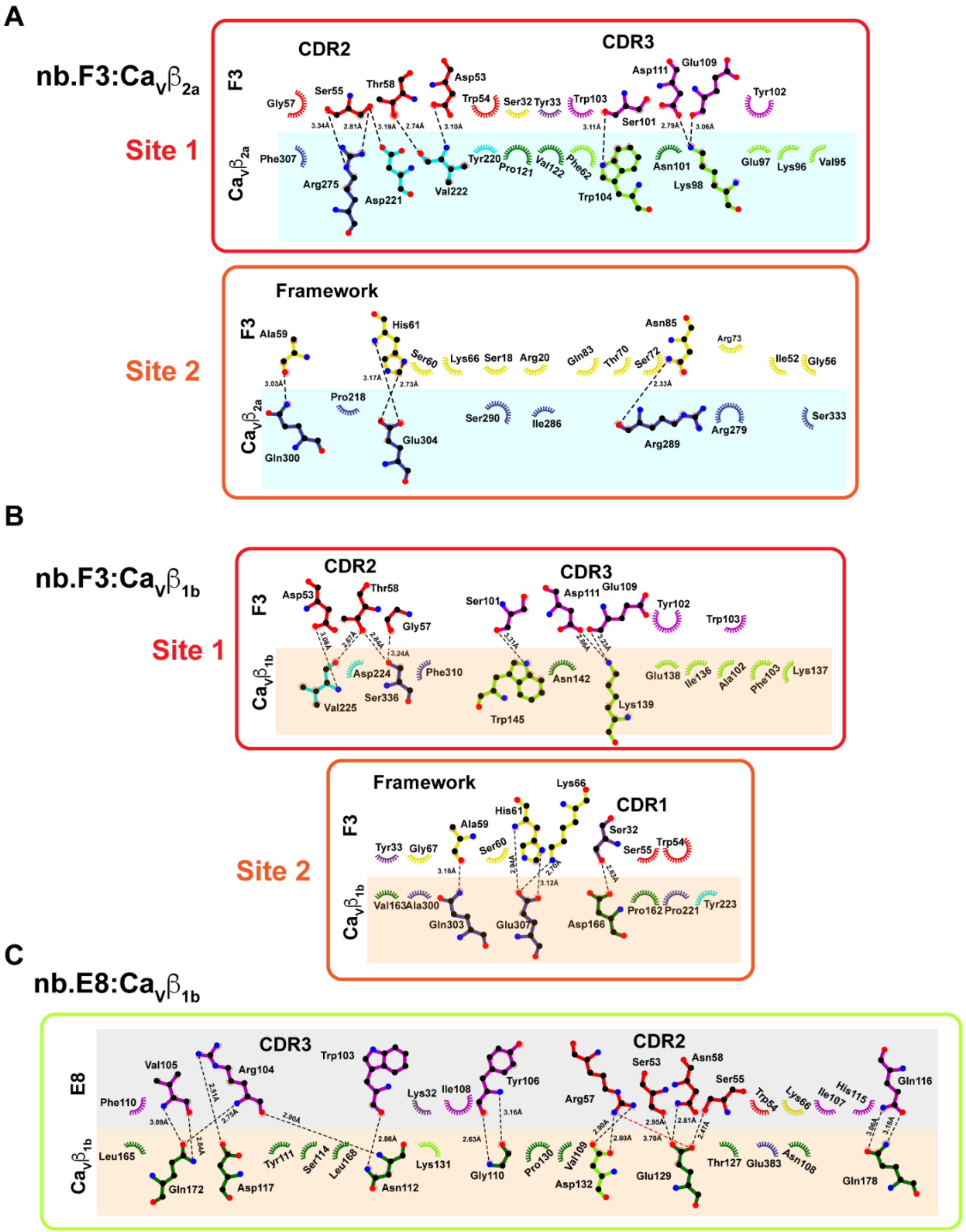

Supplementary Figure 2: nanobody:Ca$_V\beta$ contacts. Contact analysis using LIGPLOT [2] for **A,** nb.F3:Ca$_V\beta_{2a}$ **B,** nb.F3:Ca$_V\beta_{1b}$, and **C,** nb.E8:Ca$_V\beta_{1b}$ showing contacts within 5Å (coronas) and hydrogen bonds or salt bridges (dashed lines). CDR residues are colored as CDR1 (purple), CDR2 (red), and CDR3 (magenta). Framework residues are yellow. Ca$_V$ residues are colored to indicate residues from NK domain helices (dark blue) and β-strands (cyan) and SH3 domain helices (dark green) and β-strands (light green). Carbon, nitrogen, and oxygen atoms are black, blue, and red, respectively.

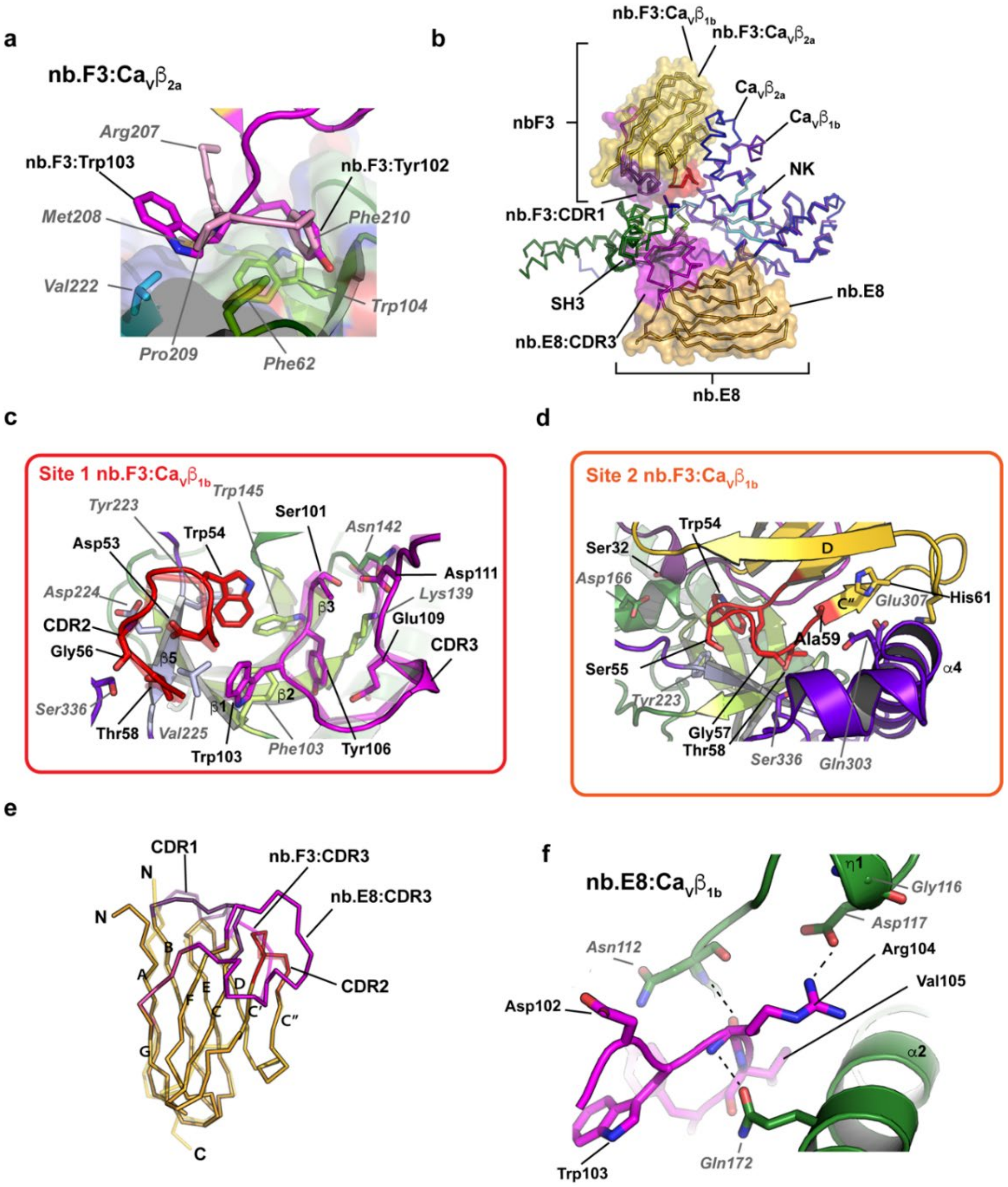

Supplementary Figure 3: Structural details of nb.F3 and nb.E8 $Ca_V\beta$ complexes. (a) Comparison of nb.F3 CDR3 (magenta) and V2 'RPMF' (pink) (PDB:1TOH) [3] SH3 domain binding site. SH3 domain surface is shown. (b) Superposition of nb.F3:$Ca_V\beta_{2a}$ and nb.F3:nb.E8:$Ca_V\beta_{1b}$ complexes. Colors are as in Fig. 3(a-b). $Ca_V\beta_{2a}$ and $Ca_V\beta_{1b}$ labels point to differences in the conformation of the 275-295 loop. (c) View of nb.F3:$Ca_V\beta_{1b}$ Site 1 interactions. (d) View of nb.F3:$Ca_V\beta_{1b}$ Site 2 interactions. (e) Superposition of nb.F3 (semi-transparent) and nb.N8 structures. CDR1 (purple), CDR2 (red), CDR3 (magenta). Nanobody residues are in black, $Ca_V\beta$ residues are labeled in grey italics. (f) Interaction of nb.E8 CDR3 (magenta) and $Ca_V\beta_{1b}$ (green) showing the molecular determinant of nb.E8 $Ca_V\beta$ isoform specificity. Dashed lines show hydrogen bonds and salt bridge. CDRs are CDR1 (purple), CDR2 (red), and CDR3 (magenta). In (a-d) and (f) nanobody residues are in black, $Ca_V\beta$ residues are labeled in grey italics.

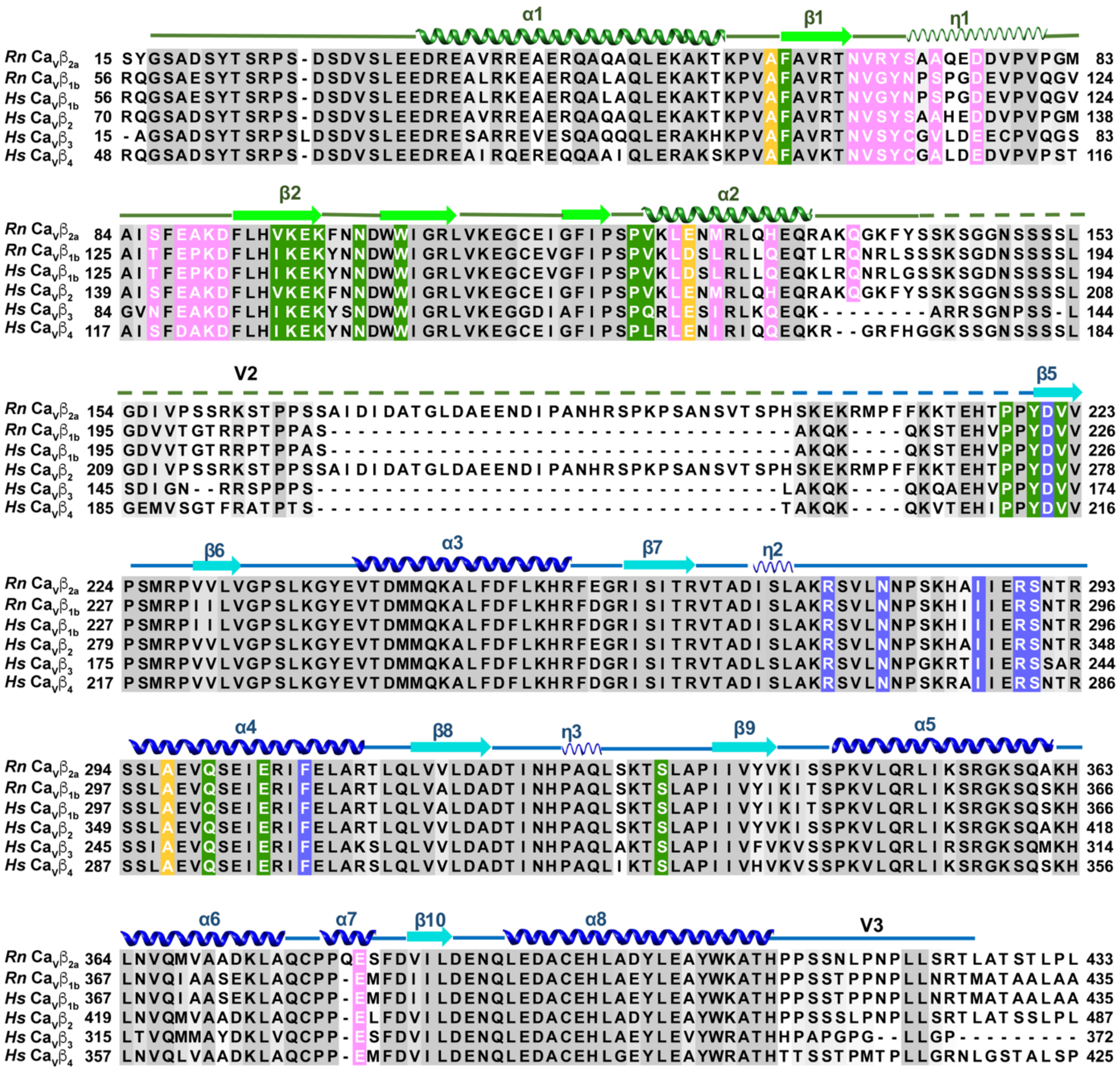

Supplementary Figure 4: Ca$_V$β sequence comparisons. Multiple sequence alignment of different Cavβs annotated with ribbon diagrams of the corresponding secondary structures. SH3 domain secondary structures are shown in green. NK domain elements are labelled in blue. Dashed lines represent sequences not present in the nb.F3-Cavβ$_{2a}$ crystal structure. V2 and V3 are variable regions which are not built due to lack of electron density. Green highlights residues common to the nb.F3-Cavβ$_{2a}$ and nb.F3-Cavβ$_{1b}$ interfaces. Cavβ$_{2a}$ residues that interact with nb.F3 are highlighted in purple. Cavβ$_{1b}$ residues that interact with nb.F3 are highlighted in yellow. Cavβ1b residues that interact with nb.E8 are highlighted in pink. Unmarked terminal residues are absent from the Cavβ$_{2a}$ and Cavβ$_{1b}$ constructs.

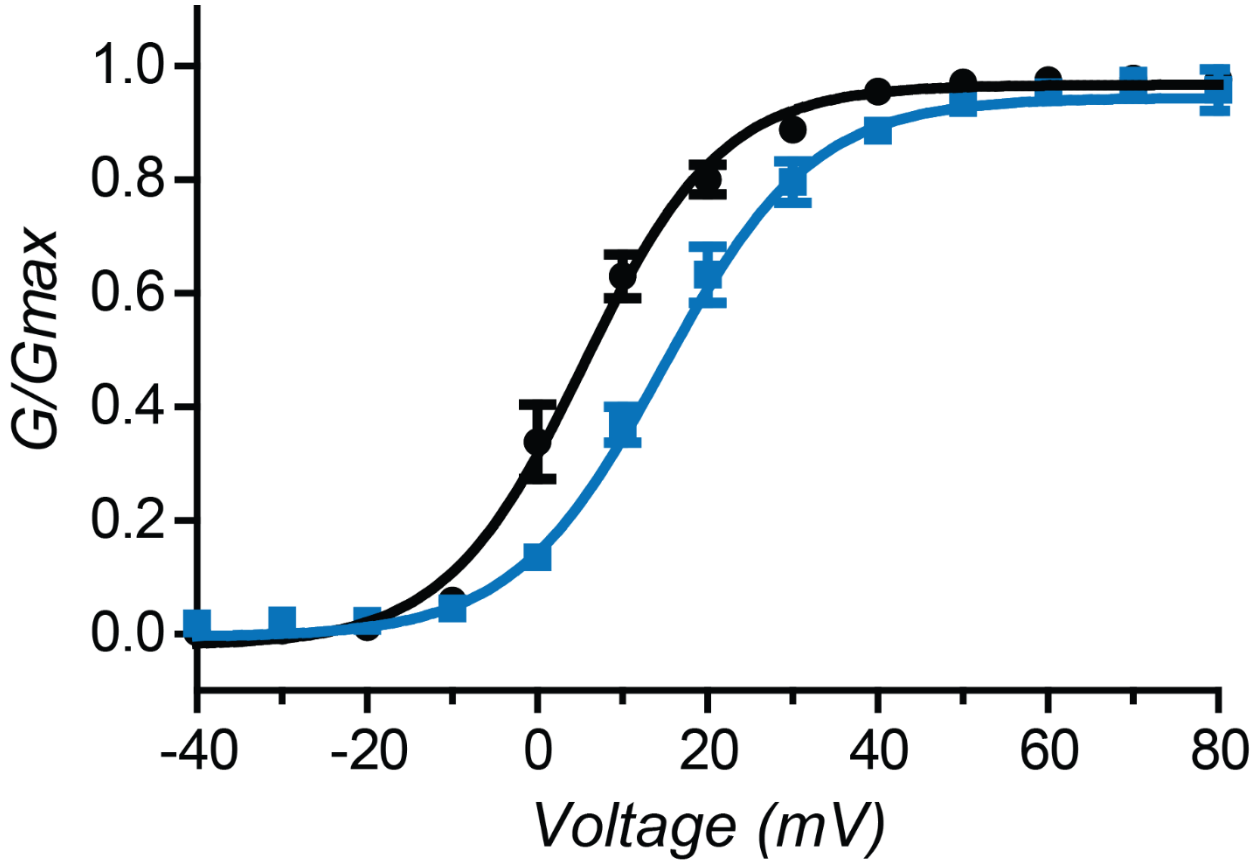

Supplementary Figure 5: Normalized ionic conductance vs voltage (*G*-*V*) relationships for cells expressing α_{1B}+β_{1b}+$\alpha_2\delta$-1 in the absence (black symbols; n = 18 from 3 independent experiments) or presence (cyan symbols; n = 10 from 3 independent experiments) of nb.E8. Data are means ± SEM. Source data are provided as a Source Data file.

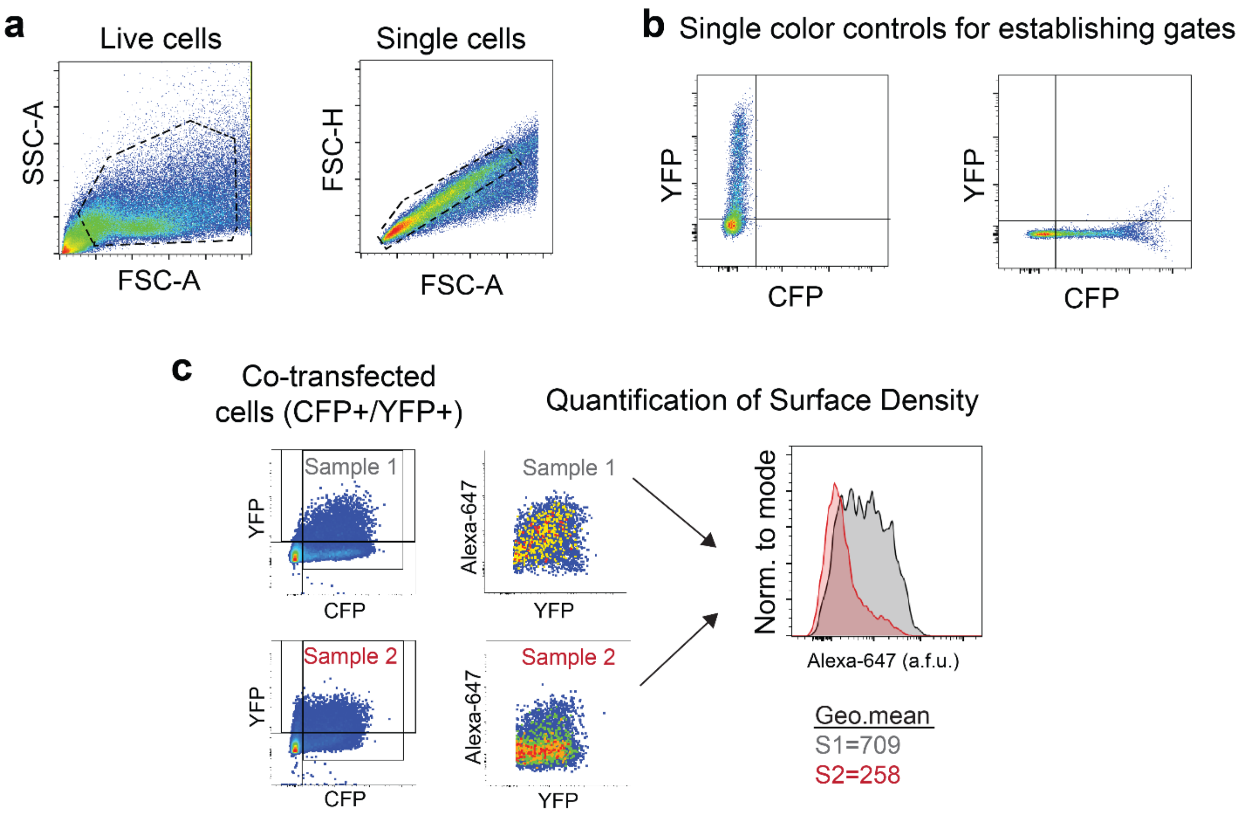

Supplementary Figure 6: Gating strategy for flow cytometry experiments. Flow cytometry pseudocolor dot plots displaying (a) an initial selection gate (*left*) for live cells (vs debris) and a second gate (*right*) for singlets (vs doublets). (b) Single fluorescent controls after applying the gating strategy in (a). (c) Exemplar analysis in which (*left*) double positive CFP+/YFP+ cells are selected for (*right*) surface density quantification (measured by BTX-Alexa-647), represented with a histogram. Sample 1 is exemplified by α1B-BBS + β1-YFP + α2δ + CFP. Sample 2 is represented by α1B-BBS + β1-YFP + α2δ + E8-Nedd4L-p2a-CFP.

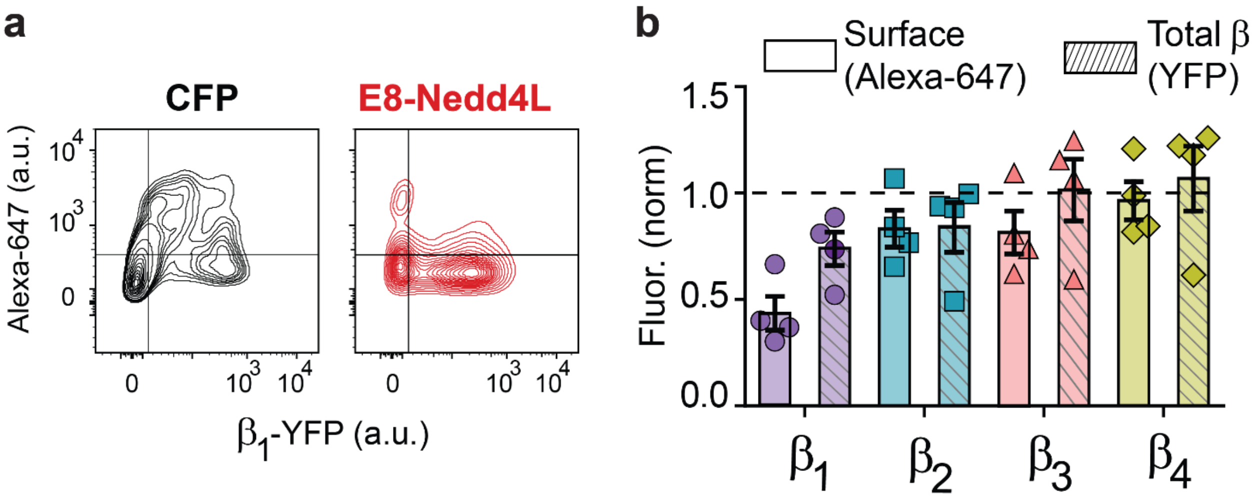

Supplementary Figure 7: (a) Flow cytometry contour plots of channel surface density (Alexa-647) versus β_{1b} expression (YFP fluorescence) in cells expressing BBS-α_{1B} + β_{1b} + $\alpha_2\delta$-1 and either CFP (control, left) or Chisel-1 (right). (b) Impact of Chisel-1 on channel surface expression and $Ca_V\beta$ expression in cells expressing BBS-α_{1B} reconstituted with distinct YFP-tagged $Ca_V\beta$ isoforms: β_{1b} (surface, n = 4; total, n = 4); β_{2b} (surface, n = 4; total, n = 4); β_3 (surface, n = 4; total, n = 4); β_4 (surface, n = 4; total, n = 4). Data are means ± SEM. Source data are provided as a Source Data file.

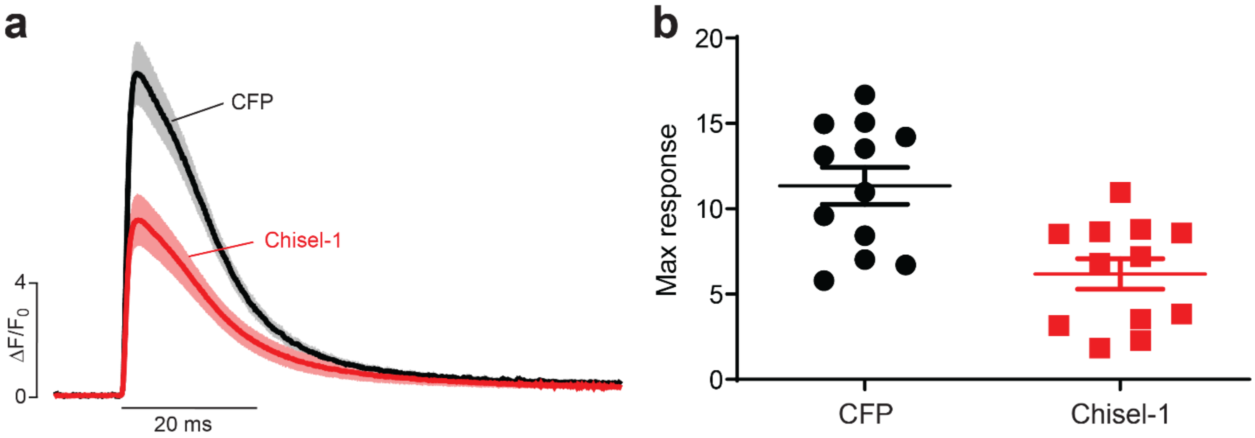

Supplementary Figure 8: Chisel-1 lowers depolarization-evoked Ca^{2+} release in skeletal muscle fibers. (a) Average Ca2+ transient waveforms evoked by external stimulation in skeletal muscle fibers expressing either CFP (black trace) or Chisel-1 (red trace). (b) Maximum evoked Ca2+ transient amplitudes in skeletal muscle fibers expressing either CFP (black circles; n = 12) or Chisel-1 (red squares; n = 12). Data are means ± SEM. Source data are provided as a Source Data file.

**Supplementary Table 1** Crystallographic data collection and refinement statistics		
	**nbF3:Cavβ_{2a}**	**nbF3:nbE8:Cavβ_{1b}**
**Data Collection**		
Space group	$P6_5$	$P2_12_12_1$
Cell dimensions a/b/c (Å)	144.54/144.54/63.6	80.358/81.072/93.332
α/β/γ (°)	90/90/120	90/90/90
Resolution (Å)	44.61 - 2.0 (2.072 - 2.0)	46.67 - 2.0 (2.072 - 2.0)
Rmerge (%)	7.188 (212.4)	10.86 (129.9)
I / σ I	32.39 (1.74)	17.53 (2.24)
CC(1/2)	1 (0.787)	0.999 (0.813)
Completeness (%)	99.85 (100.00)	98.89 (99.40)
Redundancy	20.2 (19.2)	12.7 (11.9)
Total reflections	1039234 (98252)	532477 (49120)
Unique reflections	51391 (5112)	41850 (4114)
Wilson B-factor	39.03	34.77
Wavelength (Å)	1.115830	1.1159
**Refinement**		
R_{work} / R_{free} (%)	20.87/22.71	18.73/22.61
No. of chains in AU	2	3
No. of protein atoms	3496	4236
No. of ligand atoms	57	33
No. of water atoms	210	199
RMSD bond lengths (Å)	0.004	0.003
RMSD angles (°)	0.66	0.67
Ramachandran favored/allowed/outliers (%)	98.15/1.85/0.00	98.67/1.33/0.00

References

1 Findeisen, F. *et al.* Stapled Voltage-Gated Calcium Channel (CaV) alpha-Interaction Domain (AID) Peptides Act As Selective Protein-Protein Interaction Inhibitors of CaV Function. *ACS Chem Neurosci* **8**, 1313-1326, doi:10.1021/acschemneuro.6b00454 (2017).

2 Wallace, A. C., Laskowski, R. A. & Thornton, J. M. LIGPLOT: a program to generate schematic diagrams of protein-ligand interactions. *Protein Eng* **8**, 127-134 (1995).

3 Van Petegem, F., Clark, K. A., Chatelain, F. C. & Minor, D. L., Jr. Structure of a complex between a voltage-gated calcium channel beta-subunit and an alpha-subunit domain. *Nature* **429**, 671-675, doi:10.1038/nature02588

nature02588 [pii] (2004).
