# Supplementary material for: Selective posttranslational inhibition of CaVβ1-associated voltage-dependent calcium channels with a functionalized nanobody
Source: Nat Commun. 2022 Dec 9;13:7556. doi: 10.1038/s41467-022-35025-7 (PMC9734117; doi:10.1038/s41467-022-35025-7)
Supplement: Supplementary file 3 — Reporting Summary [file 41467_2022_35025_MOESM3_ESM.pdf]

nature portfolio

Corresponding author(s): Henry M. Colecraft

Last updated by author(s): Oct 31, 2022

Reporting Summary

Nature Portfolio wishes to improve the reproducibility of the work that we publish. This form provides structure for consistency and transparency in reporting. For further information on Nature Portfolio policies, see our Editorial Policies and the Editorial Policy Checklist.

Statistics

For all statistical analyses, confirm that the following items are present in the figure legend, table legend, main text, or Methods section.

n/a	Confirmed	
☐	☒	The exact sample size (*n*) for each experimental group/condition, given as a discrete number and unit of measurement
☐	☒	A statement on whether measurements were taken from distinct samples or whether the same sample was measured repeatedly
☐	☒	The statistical test(s) used AND whether they are one- or two-sided<br>*Only common tests should be described solely by name; describe more complex techniques in the Methods section.*
☐	☒	A description of all covariates tested
☐	☒	A description of any assumptions or corrections, such as tests of normality and adjustment for multiple comparisons
☐	☒	A full description of the statistical parameters including central tendency (e.g. means) or other basic estimates (e.g. regression coefficient) AND variation (e.g. standard deviation) or associated estimates of uncertainty (e.g. confidence intervals)
☐	☒	For null hypothesis testing, the test statistic (e.g. *F*, *t*, *r*) with confidence intervals, effect sizes, degrees of freedom and *P* value noted<br>*Give P values as exact values whenever suitable.*
☒	☐	For Bayesian analysis, information on the choice of priors and Markov chain Monte Carlo settings
☒	☐	For hierarchical and complex designs, identification of the appropriate level for tests and full reporting of outcomes
☒	☐	Estimates of effect sizes (e.g. Cohen's *d*, Pearson's *r*), indicating how they were calculated

Our web collection on statistics for biologists contains articles on many of the points above.

Software and code

Policy information about availability of computer code

Data collection	Flow cytometry: BD FACSDiva (v8.01)<br>Confocal: NIS-Elements (v5.02)<br>EPhys: HEKA Pulse (v8.65); Clampex (v11)
Data analysis	FlowJo (vl0.6.0); FIJl/lmageJ (2.0.0-rc-69/1.Slo); GraphPad Prism 7; Microsoft Excel (v16.27); Origin (v9.7); PyMOL (v2.3.3); HEKA FITMaster (v2x73.5),custom Matlab software (v2012b) for FlowFRET (adapted from Lee et al. 2016, https://github.com/manubenjohny/FACS_FRET) and single-channel analysis (https://github.com/manubenjohny/SinglesAnalysis/); Clampfit (v11; Molecular Devices); XDS (general public license version 2), AIMLESS (CCP4Interface 8.0.000), PHASER (part of PHENIX suite version 1.20.1-4487-000), COOT (v0.9.8.1EL), PHENIX (v1.20.1-4487-000

For manuscripts utilizing custom algorithms or software that are central to the research but not yet described in published literature, software must be made available to editors and reviewers. We strongly encourage code deposition in a community repository (e.g. GitHub). See the Nature Portfolio guidelines for submitting code & software for further information.

Data

Policy information about availability of data

All manuscripts must include a data availability statement. This statement should provide the following information, where applicable:

- Accession codes, unique identifiers, or web links for publicly available datasets
- A description of any restrictions on data availability
- For clinical datasets or third party data, please ensure that the statement adheres to our policy

The authors declare that all data supporting the findings of this study are available within the paper and its supplementary information files. Coordinates and structure factors for the CaVβ1b:nb.E8 (PDB:8DAM) and CaVβ2a:nb.F3 (PDB:8E0E) complexes are deposited with the RCSB. PDB:5V2P is available at RCSB PDB [https://www.rcsb.org/structure/5v2p] (CaVβ2a:CaV1.2 AID peptide complex). The source data underlying Figure 2 (a,b,i), Figure 4 (b,c,e,f,h,i,k,l), Figure 5 (c,g), Figure 6 (c-f,h-k), Figure 7 (a-c), Figure 8 (c,d,g,h), Figure 9 (b,d,f), Supplementary Figure 5, Supplementary Figure 7b, and Supplementary Figure 8b are provided as a Source Data File.

Human research participants

Policy information about studies involving human research participants and Sex and Gender in Research.

Reporting on sex and gender	The study included no human research
Population characteristics	The study included no human research
Recruitment	The study included no human research
Ethics oversight	The stud included no human research

Note that full information on the approval of the study protocol must also be provided in the manuscript.

Field-specific reporting

Please select the one below that is the best fit for your research. If you are not sure, read the appropriate sections before making your selection.

☒ Life sciences ☐ Behavioural & social sciences ☐ Ecological, evolutionary & environmental sciences

For a reference copy of the document with all sections, see nature.com/documents/nr-reporting-summary-flat.pdf

Life sciences study design

All studies must disclose on these points even when the disclosure is negative.

Sample size	The sample size of experiments was based on previous experience and published studies (e.g. Morgenstern et al, 2019, elife). The sample sizes are robust enough based on the magnitude of the observed differences and consistency of the various measurements.
Data exclusions	For whole-cell electrophysiological experiments cells were excluded if the seal resistance <200 Mohm resulting in a large basal leak current (heterologous cells and ventricular cardiomyocytes). These are pre-established criteria from years of experience with electrophysiological experiments.
Replication	Experiments had at least 3 independent successful replicates.
Randomization	No randomization was applied based on the types of experiments. Test and control groups were assessed in parallel and isochronally.
Blinding	No blinding was applied due to the nature of the experiments. The most effective process was for one experimentalist to prepare the cells and conduct the experiments. As such, they were aware of the different control and test conditions.

Reporting for specific materials, systems and methods

We require information from authors about some types of materials, experimental systems and methods used in many studies. Here, indicate whether each material, system or method listed is relevant to your study. If you are not sure if a list item applies to your research, read the appropriate section before selecting a response.

Materials & experimental systems

n/a	Involved in the study	
	x	Antibodies
	x	Eukaryotic cell lines
x		Palaeontology and archaeology
	x	Animals and other organisms
x		Clinical data
x		Dual use research of concern

Methods

n/a	Involved in the study	
x		ChIP-seq
	x	Flow cytometry
x		MRI-based neuroimaging

Antibodies

Antibodies used	Rabbit anti-beta1(Alomone Labs, Cat#ACC-106, polyclonal, WB 1:1000 dilution, IP 0. 75µg); Rabbit anti-beta2 (Alomone Labs, Cat# ACC-105, WB 1:1000 dilution) ; Rabbit anti-beta3 (Alomone Labs, Cat#ACC-008, WB 1:1000 dilution); Mouse anti-beta4 (NeuroMab, Cat#75-05, WB 1:1000 dilution); Rabbit anti-Cav1.2 (Alomone Labs, Cat#ACC-003, WB 1:1000 dilution); Rabbit anti-actin (Sigma Aldrich, Cat#A5060, WB 1:1000 dilution); Mouse anti-ubiquitin (LifeSensors, Cat#VU101, WB 1:500), Goat anti-Rabbit (ThermoFisher, Cat#710369, IB: 1:10,000), Goat anti-Mouse (ThermoFisher, Cat#A21236, IB: 1:10,000); MAP2 (Santa Cruz Biotechnology, Cat#sc-74421, IB: 1:1,000); Rabbit anti-pCREB (Cell Signalling Technology, Cat#9198, IB: 1:333).
Validation	All antibodies were validated by manufacturers. Rabbit anti-beta1 (alomone.com/p/anti-cav1/ACC-106?b=35967); Rabbit anti-beta2 (https://www.alomone.com/p/anti-cav2/ACC-105); Rabbit anti-beta3 (https://www.alomone.com/p/anti-cav3/ACC-008); Mouse anti-beta4 (https://neuromab.ucdavis.edu/datasheet/N10_7.pdf); Rabbit anti-Cav1.2 (https://www.alomone.com/p/anti-cav1-2-antibody/ACC-003); Rabbit anti-actin (https://www.sigmaaldrich.com/US/en/product/sigma/a5060); Mouse anti-ubiquitin (https://lifesensors.com/product/vu101-anti-ubiquitin-antibody-mab-clone-vu-1/); Goat anti-Rabbit (https://www.thermofisher.com/antibody/product/Alexa-Fluor-488-Antibody-Recombinant-Polyclonal/710369); Goat-anti Mouse (https://www.thermofisher.com/antibody/product/Goat-anti-Mouse-IgG-H-L-Highly-Cross-Adsorbed-Secondary-Antibody-Polyclonal/A-21236); Mouse anti-MAP2 (https://www.scbt.com/p/map-2-antibody-a-4); Rabbit anti-pCREB (https://www.cellsignal.com/products/primary-antibodies/phospho-creb-ser133-87g3-rabbit-mab/9198?N=4294956287&Ntt=9198s&_requestid=353940&fromPage=plp&site-search-type=Products)

Eukaryotic cell lines

Policy information about cell lines and Sex and Gender in Research

Cell line source(s)	Human embryonic kidney (HEK293) cells were a kind gift from the laboratory of Dr. Robert Kass (Columbia University) and were originally obtained from ATCC.
Authentication	HEK293 cells were authenticated via STR analysis (ATCC) with exact match for ATCC CRL-1573.
Mycoplasma contamination	Cells were mycoplasma free, as determined by the MycoFluor Mycoplasma Detection Kit (Invitrogen).
Commonly misidentified lines (See ICLAC register)	No commonly misidentified cell lines were used.

Animals and other research organisms

Policy information about studies involving animals; ARRIVE guidelines recommended for reporting animal research, and Sex and Gender in Research

Laboratory animals	Adult male Hartley guinea pigs (Charles River) , 250 g, 4 weeks old; Adult male C57BL/6J mice (Charles River)
Wild animals	No wild animals were used in the study.
Reporting on sex	We isolated cardiomyocytes from adult male Hartley guinea pigs.
Field-collected samples	No field-collected samples were used in the study.
Ethics oversight	Experiments using guinea pigs were performed in accordance with the guidelines of Columbia University Animal Care and Use Committee (IACUC). Experiments in mice were performed according to protocols approved by the University of Maryland Institutional Animal Care and Use Committee

Note that full information on the approval of the study protocol must also be provided in the manuscript.

Flow Cytometry

Plots

Confirm that:

- [x] The axis labels state the marker and fluorochrome used (e.g. CD4-FITC).
- [x] The axis scales are clearly visible. Include numbers along axes only for bottom left plot of group (a 'group' is an analysis of identical markers).
- [x] All plots are contour plots with outliers or pseudocolor plots.
- [x] A numerical value for number of cells or percentage (with statistics) is provided.

Methodology

Sample preparation	Samples were prepared as described in our methods section (flow cytometry).
Instrument	BD LSRII Cell Analyzer (BD Biosciences, San Jose, CA, USA)
Software	BD FACSDiva was used to collect data. FlowJo (vl0.6.0) was used to analyze surface labeling assays. A custom Matlab software was used for flow cytometric FRET analysis (adapted from Lee et al 2016, https://github.com/manubenjohny/FACS_FRET)
Cell population abundance	N/A; HEK293 cells were not sorted prior to our flow cytometric analyses, but rather transiently transfected.
Gating strategy	Appropriate gates were used to determine cells vs debris (FSC/SSC) and single cells vs doublets (FSC-A/FSC-H). Subsequent gates were determined with single color controls, and values were analyzed from co-transfected cell populations (YFP- and CFP-positive as described in our manuscript). The gating strategy is indicated in Supplementary Figure 6.

- [x] Tick this box to confirm that a figure exemplifying the gating strategy is provided in the Supplementary Information.
